# Modulating the Gut Micro-Environment in the Treatment of Intestinal Parasites

**DOI:** 10.3390/jcm5110102

**Published:** 2016-11-16

**Authors:** Luis Vitetta, Emma Tali Saltzman, Tessa Nikov, Isabelle Ibrahim, Sean Hall

**Affiliations:** 1 Sydney Medical School, The University of Sydney, Sydney 2006, NSW, Australia; esal4025@uni.sydney.edu.au; 2 Medlab Clinical Ltd., Sydney 2015, NSW, Australia; tessa_nikov@medlab.co (T.N.); isabelle_ibrahim@medlab.co (I.I.); sean_hall@medlab.co (S.H.)

**Keywords:** probiotics, protozoans, giardiasis, blastocystosis, intestinal dysbiosis

## Abstract

The interactions of micro-organisms cohabitating with *Homo sapiens* spans millennia, with microbial communities living in a symbiotic relationship with the host. Interacting to regulate and maintain physiological functions and immunological tolerance, the microbial community is able to exert an influence on host health. An example of micro-organisms contributing to an intestinal disease state is exhibited by a biodiverse range of protozoan and bacterial species that damage the intestinal epithelia and are therefore implicated in the symptoms of diarrhea. As a contentious exemplar, *Blastocystis hominis* is a ubiquitous enteric protist that can adversely affect the intestines. The symptoms experienced are a consequence of the responses of the innate immune system triggered by the disruption of the intestinal barrier. The infiltration of the intestinal epithelial barrier involves a host of immune receptors, including toll like receptors and IgM/IgG/IgA antibodies as well as CD8+ T cells, macrophages, and neutrophils. Whilst the mechanisms of interactions between the intestinal microbiome and protozoan parasites remain incompletely understood, it is acknowledged that the intestinal microbiota is a key factor in the pathophysiology of parasitic infections. Modulating the intestinal environment through the administration of probiotics has been postulated as a possible therapeutic agent to control the proliferation of intestinal microbes through their capacity to induce competition for occupation of a common biotype. The ultimate goal of this mechanism is to prevent infections of the like of giardiasis and eliminate its symptoms. The differing types of probiotics (i.e., bacteria and yeast) modulate immunity by stimulating the host immune system. Early animal studies support the potential benefits of probiotic administration to prevent intestinal infections, with human clinical studies showing probiotics can reduce the number of parasites and the severity of symptoms. The early clinical indications endorse probiotics as adjuncts in the pharmaceutical treatment of protozoan infections. Currently, the bar is set low for the conduct of well-designed clinical studies that will translate the use of probiotics to ameliorate protozoan infections, therefore the requisite is for further clinical research.

## 1. Introduction

Decades of research has revealed that the prevalence, persistence, and diversity of bacteria has been consistently undervalued [1] with life on this planet running concurrently with bacterial interactions at all levels of the living environment. Bacteria inhabit all manners of ecosystems including those found beneath the ice sheets in Antarctica [2] that contribute to sub-glacial aqueous environments, to those found in diverse geochemical environments of Yellowstone that host a variety of chemotrophic and phototrophic thermophilic communities [3]. It is therefore concluded that microbial communities form most of the world’s biomass [4]. Reported estimates of bacterial mass suggest that there is approximately 40 million bacterial cells per gram of soil and one million bacterial cells per gram (mL) of fresh water [4]. The human microbiome project reports that 90% of the cells in the human body belong to bacteria, fungal, or other non-human (e.g., parasites) elements [5] that can influence the host’s health or disease state. Axenic experiments with rodents [6] have demonstrated that the microbiome community living on and within the mammalian host is able to shape its nutrient environment. A diverse and multifaceted environment emerges where, through the microbial interactions in the intestine, the gut microbiome is able to instruct the host in the complex language of molecular biology [7], allowing it to affect the pathogenesis of disease and infection.

There is a wide range of protozoans that are parasites of the human intestines. These protozoan species are not classified as a homogenous group. The physiology and biochemistry of human intestinal parasites are largely geared toward a parasitic habit [8]. The mechanisms of host penetration varies according to protozoan type, namely some species are intracellular parasites (e.g., *Cryptosporidium* spp.) and host specialized (e.g., *Entamoeba histolytica*), whereas others adapt to more than one host (e.g., *Giardia intestinalis*). Few species are reported to do any real damage to the host but some species can occasionally lead to common symptoms of diarrhea due to damage of the intestinal lining of the gut.

Studies report that protozoan pathogenesis largely results from parasitic biochemical products (e.g., proteinases) that can penetrate and damage the intestinal epithelial barrier, adversely affecting host inflammatory and immunological responses, as has been observed for *Giardia* [9] and *Cryptosporidium* [10]. The recognition of protozoans parasitizing mucosal surfaces in the intestines may involve the innate immune system/toll like receptors (TLRs) as shown through in vivo observations in individuals infected with *Trichomonas vaginalis* [11]. Furthermore, it has been reported that CD8+ T cells, macrophages, neutrophils, and IgM/IgG/IgA antibodies are major participants of the acquired immune response that is required for the resolution of giardiasis [8].

Although the pathogenicity of *Blastocystis hominis* is contentious, we propose that it forms part of the environment in the intestines of infected individuals. In fact, *B. hominis* is probably the most common protozoan detected in human faecal samples worldwide. Infection occurs in both immune-competent and immune-compromised individuals. Reported prevalence ranges from 2%–50% with the highest rates reported for developing countries with poor environmental hygiene. Infection appears to be more common in adults than in children [12,13].

The use of probiotics to modulate the gastrointestinal microbiome has been posited as a potential novel therapeutic agent [14]. It is hypothesized that probiotics have the capacity to control the proliferation of intestinal microbes by initiating competition for occupation of a common ecological niche. This mechanism aims to prevent protozoan infections of the like of *Giardiasis* and eliminate its symptoms. One of the many functions of the varying types of probiotics, including bacteria and yeasts, is to regulate immunity by stimulating the immune system within the host. Murine model studies (Table 1) support the use of probiotic administration as a therapeutic agent to prevent intestinal infections. Clinical studies (Table 2) have shown that probiotics can reduce the number of parasites and severity of associated symptoms. As such, early clinical indications support the use of probiotics as therapeutic adjuncts in the pharmaceutical treatment of protozoan infections.

## 2. Methodology

A systematic search of the literature was conducted using PubMed, the Cochrane Library, and CINAHL, as well as bibliographies of past research on the subject. Search terms were not limited by timeframe and therefore all searches were between July 2016 and the date of inception for each database. Articles were identified using the search terms (“*Protist*” OR “intestinal protozoan”) AND (“dysbiosis”) AND (“murine studies” or “human studies”) AND (“probiotics” OR “pharmaceuticals”) AND (“*Lactobacilli*” or “*Bifidobacteria*” or “*Saccharomyces boulardii*”). Inclusion criteria for this review were: (1) RCT and/or cross-over trials that used either placebo or current anti-infective medication or probiotics treatments; (2) Animal studies limited to probiotic or pharmaceutical treatments in laboratory mice or rats; and (3) articles published in English. Following the search, non-English papers were excluded from data extraction and analysis.

## 3. Intestinal Environment Interactions between the Microbiota and Protozoans

Although the interactions between the intestinal microbiome and protozoan parasites remain poorly understood [8], the intestinal microbiota represents an important actor in the pathophysiology of parasite infections.

Early murine studies have demonstrated that the intestinal microbiome could decrease susceptibility to *Cryptosporidium parvum* infections [23]. In contrast, other studies have shown that the intestinal microbiome was an essential factor for the expression of other pathogenic enteric parasites such as *B. hominis* [24] and *E. histolytica* [25]. Of significant interest has been a study on *Giardia muris* trophozoites with transmission electron microscopy [26] that showed bacterial endosymbionts within *G. muris* trophozoites have a role in both host protective and pathological mechanisms. It is speculated that this dual role is derived through altering the trophozoite antigencity. The protective role of bacterial endosymbionts within *G. muris* trophozoites was evident when trophozoites with endosymbionts were lysed when in close vicinity of the activated Paneth cells [26]. Such results support the hypothesis that the intestinal microbiota may directly and indirectly interfere with the pathogenesis of giardiasis.

In contrast, axenisation of the host at the intestinal level has been shown to promote the virulent expression of protozoan parasites. Studies with *E. histolytica* have demonstrated that interactions of amoebae of low pathogenicity with a variety of Gram-negative gut bacteria such as *Escherichia coli* strains could be responsible for the increased amoebic virulence [25]. Other studies have shown that the intestinal microbiota can stimulate the pathogenic expression but not the multiplication of parasites [27]. Torres and colleagues showed that the intestinal bacteria responsible for the stimulation of *Giardia duodenalis* pathogenicity dominate the duodenal microbiota [27]. Facultative and strictly anaerobic microbes from the duodenal microbiota were obtained from the biopsy of five children with symptomatic giardiasis. These microbes were then tested for their ability to stimulate *G. duodenalis* pathogenicity in gnotoxenic mice [27]. The germ-free animals did not develop intestinal pathological modifications during experimental *Giardia* infection, whilst further infected gnotoxenic mice showed intermediate pathological alterations between germ-free and infected conventional mice that were used as controls. No pathological changes were observed in non-infected gnotoxenic or conventional animals [27]. A hypothesis is suggested that bacterial components from the intestinal microbiota are required as stimulatory factors for intestinal pathogenic protozoans as well as for *Giardia* pathogenicity.

Modulation of the intestinal environment by probiotic bacteria can also serve as a trigger for controlling the proliferation of intestinal microbes and induces competition for the occupancy of a common biotope [28]. For example, *Lactobacilli* can limit nutrient availability. Such probiotic bacteria can render iron unavailable to pathogenic micro-organisms by either binding ferric hydroxide on its surface or by secreting siderophores (organic compounds produced by micro-organisms under conditions of low iron) that chelate and transport iron [29,30].

Some probiotics are also able to influence the composition and equilibrium of the gut microbiota [31]. For example, probiotic therapy using a multi-species probiotic formulation increased the total number of intestinal bacteria and restored the microbiota’s diversity in patients diagnosed with pouchitis [32]. *Bifidobacteria* shows efficacy in the allevation of gastrointestinal symptoms including constipation, abdominal discomfort, and flatulence through rebalancing of the gut microbiota [28]. Probiotic bacteria also exerts influence on the biotic environment by regulating the secretion of mucus and intestinal motility [28]. Probiotic bacteria can also secrete active molecules (i.e., bacteriocins, antibiotics, free fatty acids, and hydrogen peroxide [33]) that can control growth and/or survival of micro-organisms inhabiting the local intestinal areas [33]. Bacteriocins are secreted peptides or proteins that generally lyse closely related bacteria by permeabilizing bacterial membranes or by interfering with essential enzymes [31,33]. *Lactobacilli* probiotic strains produce various compounds—such as lactacin B, lactacin F, and nisin—that have bacteriocidal effects [34]. *Lactobacillus reuteri* produces reuterin (3-hydroxypropionaldehyde), a broad-spectrum antibiotic, that is active against bacteria, viruses, yeasts, fungi, and protozoa [34]. Lactic acid probiotic bacteria can also modify the growth of acid-sensitive organisms by lowering the local intestinal pH with the elaboration of lactic acid [29].

Probiotic bacteria can also modulate immunity by stimulating the host immune response to a variety of pathogens. In the intestine, probiotics interact with the epithelial cells, Peyer’s patches, M cells and immune cells [7]. These interactions result in an increase in the number of IgA producing cells accompanied by production of IgM and secretory IgA entities that are particularly important in mucosal immunity as they contribute to the barrier against pathogenic organisms [35]. In addition, probiotic bacteria can also affect dendritic cells, which are responsible for the collection of antigens from the intestine and their presentation to naive T cells which leads to their differentiation to T-helper (Th1, Th2) or T-regulatory lymphocytes. Probiotic molecules implicated in dendritic cell induction are poorly characterised with one exception being the S layer protein A of *L. acidophilus* NCFM that regulates maturation of dendritic cells and T cell functions [36].

Probiotics have also been shown to modulate cytokine release (TNF-α IFN-γ, IL-10, IL-12) [36]. Cytokine actions are important as they are central to maintaining intestinal homeostasis, the subtle balance that is required to maintain necessary and exhaustive defense mechanisms in the gut. Polysaccharide A, synthesized by the probiotic *Bacillus fragilis,* has been shown to protect against experimental colitis through a tolerable induction of IL-10 production [37].

## 4. Probiotics for the Treatment of Protozoan Infections

Intestinal protozoa can cause clinically significant infections in both the developed and developing words where the organisms are responsible for the occurrence of acute and chronic diarrhea [38]. The clinical impact of infection with intracellular intestinal protozoa (*Cryptosporidium*, *Microsporidia*, *Cyclospora*, *Isospora*) can be extremely damaging, especially in susceptible individuals who are immune incompetent. The administration of nitazoxanide (a substituted benzamide) and co-trimoxazole can effectively treat and clear Cryptosporidiosis and *Cyclospora* infections respectively [38]. However, the addition of these medications risks damaging the intestinal mucosa and micro-organism environment by disrupting the balance of the microbial composition [39].

Numerous laboratory animal studies (Table 1) have demonstrated that probiotic bacteria, such as the administration of *Lactobacillus johnsonii* [40] on *G. lambia* cysts in the feces of gerbils or the addition of *Lactobacillus casei* [15] as well as *Enterococcus faecium* (a species commonly found in the human gut microbiota) [41], have been able to effectively eliminate *Giardia* infections from mice. Mice that maintained an intact and competent immune system through probiotic treatment were refractory to *C. parvum*, with a compromised immune system altering cytokine production and leading to persistent cryptosporidiosis. Murine model studies have also demonstrated that *L. reuteri* supplementation can help prevent *C. parvum* [16,17]. These animal studies confirm that probiotics may confer benefits that strengthen host resistance to intestinal parasitic infections.

An early clinical study (Table 2) demonstrated that the beneficial effect of the administration of the probiotic yeast *Saccharomyces boulardii* is not directly related to its ability to prevent giardiasis but rather its capacity to assist with the recovery from post infection irritable bowel syndrome [42]. A later study from another group supported the earlier report by showing that *S. boulardii* reduced the number of parasite cysts in the feces from patients treated with the combination treatment of *S. boulardii* plus metronidazole versus patients treated by metronidazole only [18]. An additional clinical study that administered *S. boulardii* or metronidazole showed potential beneficial effects in *B. hominis* infection relative to a reduction of symptoms and presence of parasites [19].

## 5. Discussion

Intestinal microbial dysbiosis has been observed in extra-intestinal diseases and in particular those that may impact on the intestine–kidney axis or the intestine–liver axis to affect the adverse changes in end organ physiology [43]. Various factors influence the integrity of the intestinal barrier such as changes in intestinal permeability, mucin production and composition, and the homeostatic balance between the rate of apoptosis of damaged enterocytes to the rate of production of new enterocytes in the intestine [43].

It is reported that the mucosa of the small intestine is regenerated in its entirety every three to five days through the continuous cycle of proliferation, migration, and differentiation of the cells of the mucosa over the entire crypt–villus axis in the intestine [44]. This mucosal life cycle has led to the probing of probiotic bacteria and the yeast *Saccharomyces boulardii* as potential adjunct pharmaceutical treatments for intestinal protozoan infections that protect the intestinal epithelia from disruptive dysbiotic effects.

**Figure 1:**
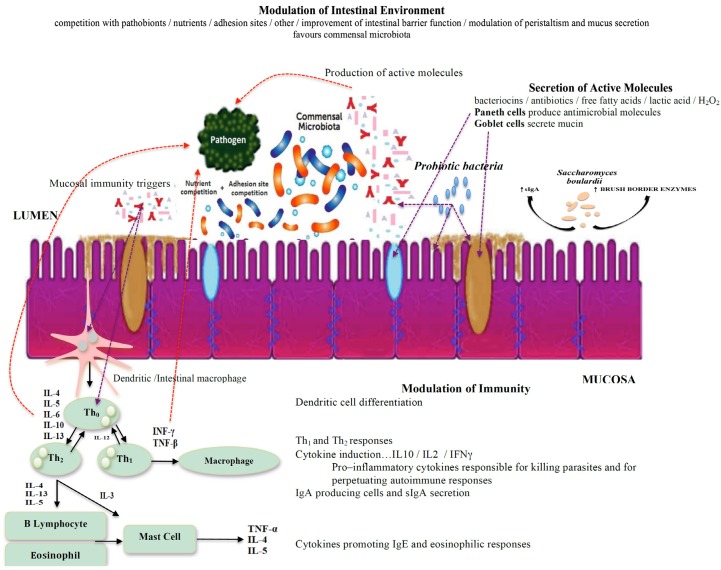
Schematic presentation of commensal, pathobiont, probiotic bacteria, and yeast interactions in the intestines with the production of antimicrobial compounds. Adapted from [47,48,49].

Protist infections with protozoans such as *Blastocystis* can alter human intestinal epithelial permeability by damaging the epithelia [45]. Animal and human studies that administered probiotic species to eradicate intestinal protozoans have demonstrated that probiotic bacteria and *S. boulardii* can modulate the intestinal environment by influencing local mucosal activity [46]. The effects of probiotic administration include a beneficial boost in T cells and the induction of immune-modulating proteins such as sIgA and a series of cytokines (Figure 1).

Furthermore, recent evidence suggests that the products of commensal intestinal microbes can help counter adverse inflammatory diseases in the host. Commensal bacteria in the colon are known to ferment dietary fibres to produce short-chain fatty acids. A mouse model from a colitis study demonstrated that these fatty acids downregulate innate and inflammatory responses through the stimulation of the chemo-attractant receptor GPR43 on neutrophils [50]. This activity strongly identifies interactions between short-chain fatty acids and GPR43 as a possible target for the manipulation of immune responses for therapeutic purposes. Moreover, the probiotic battery of multiple mechanisms for preventing infections, enhancing the immune system and providing immunomodulatory benefits includes an array of anti-microbial compounds that are capable of inhibiting the growth of pathogens, irrespective of the type of micro-organism that may overgrow or infect the intestines; namely bacteria, protozoans, and opportunistic pathogenic yeasts [51].

Probiotics are often classified as supplements of viable non-pathogenic micro-organisms (i.e., bacteria or yeast) that when orally administered can confer a health benefit to the host. Bacterial species of probiotics include *Lactobacilli* species, *Bifidobacteria* species, some *Escherichia coli* species, *Streptococcus* species, and the yeast *Saccharomyces boulardii* [52]. Herewith, we posit that probiotics are not supplements but rather medicines that confer positive health benefits through the modulation of the intestinal micro-environment [7]. Probiotic bacteria from the genus *Lactobacilli* and *Bifidobacteria* have been reported to contribute to a decrease in luminal pH, stimulate epithelial cell growth and colonic blood flow, modify intestinal motility and absorb water and minerals, and increase the production of mucus [47].

## 6. Conclusions

It is evident that the microbial community of the human intestine is in a state of continuous flux, with the intestinal microbiome of a healthy adult composed of a stable balanced community of micro-organisms [53]. Comprising an ecosystem of living cells that interact with each other, an array of molecular compounds are produced which fine-tune the language of molecular biology in the intestine between micro-organisms and host cells that serve to maintain health or trigger disease. A symbiotic relationship therefore exists between the mammalian host and its intestinal microbiome. The disruption of this symbiotic relationship, termed dysbiosis, is the link used to implicate the intestinal microbiome in the pathogenesis of both disease and infection in the host. However, further research is warranted to clarify the mechanisms of action that lead the microbiome to initiate parasitic infections.

Modulating the intestinal microbial balance to restore balance is proposed as a possible therapeutic adjunct to attenuate or treat certain parasitic infections. One means of restoring homeostasis to the intestinal environment is the supplementation of probiotics. This review summarized animal studies and early clinical trials that administered probiotics and the yeast *Saccharomyces boulardii* to treat parasitic infections, with different probiotic strains, with *S. boulardii* having shown the capacity to reduce the symptoms of diarrheal infections and assist with recovery.

## Figures and Tables

**Table 1:** Laboratory animal studies on the administration of probiotics to eradicate protist infections.

Reference	Year	Study	Methods	Results	Conclusion
[15]	2008	Effect of *Lactobacillus casei* as a probiotic on modulation of giardiasis	Group I = single dose of TYI-*S*-33 Group II = *Giardia*-infected mice—single dose of *Giardia* trophozoites qid. Group III = probiotic group IIIa: *L. casei* qid. for 30 days IIIb: isolated strain A (containing lactobacilli) qid. for 30 days Group IV = *Giardia*-probiotic IV a: single dose of *Giardia* trophozoites & *L. casei* for 30 days. IV b: single dose of *Giardia* trophozoites and a single dose of isolated strain A od. for 30 days Group V = probiotic-*Giardia*—Probiotic treatment for 30 days after infective dose of *G. lamblia*. Va: single dose of *L. casei* qid. for seven days then a single infective dose of *Giardia* trophozoites. Vb: single dose of isolated strain A qid. for seven days then a single infective dose of *Giardia* trophozoites.	*L. casei* given seven days prior to *Giardia* infection more effective and efficient in eliminating infection Probiotic-fed mice had less atrophied villi and infiltrating cells in the small intestine Ultrastructural studies with scanning electron microscopy confirmed protection of mice receiving *L. casei* seven days prior to *Giardia* infection and when simultaneously infected with *Giardia*.	Probiotics, particularly *L. casei*, modulate *Giardia* infection by minimising or preventing adherence of *Giardia* trophozoites to the mucosal surface, suggesting probiotics offer a safe and effective mode to prevent and treat *Giardia* infection.
[16]	1997	Effect of *L. reuteri* on intestinal resistance to *C. parvum* infection in acquired immunodeficient murine model	C57BL/6 immunosuppressed female mice with LP-BM5 leukemia virus Four months after inoculation, mice developed susceptibility to *C. parvum* infection. Daily prefeeding with *L. reuteri* (10^8^ cfu/day) for 10 days then challenged with 6.5 × 10^6^ *C. parvum* oocysts Both groups fed *L. reuteri* for duration of study.	Supplemented mice cleared parasite loads from the gut epithelium. Control mice developed persistent cryptosporidiosis,shed high levels of oocysts in faeces and increased colonization of the intestinal tract	*L. reuteri* may help prevent *C. parvum* infection in immune-deficient subjects.
[17]	1999	Supplementation with *Lactobacillus reuteri* or *L. acidophilus* reduced intestinal shedding of *cryptosporidium parvum* oocysts in immunodeficient C57BL/6 mice.	C57BL/6 immunosuppressed female mice with LP-BM5 leukemia virus and randomly assigned to one of five groups Group A: historical control Group B: LP-BM5 control Group C: *C. parvum* Group D: *L. reuteri* plus *C. parvum* Group E: *L. acidophilus* plus *C. parvum* Mice pre-fed with *L. reuteri* or *L. acidophilus* for 13 days then challenged with *C. parvum* oocysts and thereafter followed their allocated prescription.	Mice supplemented with *L. reuteri* shed fewer (*p* < 0.05) oocysts on Day 7 post *C. parvum* challenge Mice supplemented with *L. acidophilus* shed fewer (*p* < 0.05) oocysts on days 7 and 14 post-challenge Lactobacillus supplementation reduced *C. parvum* shedding in the feces but failed to suppress production of IL-4 & IL-8 & restore IL-2 & IFN-gamma	*L. reuteri* or *L. acidophilus* can reduce *C. parvum* parasite burdens in epithelium during cryptosporidiosis. *L. acidophilus* was more efficacious in reducing fecal shedding than *L. reuteri*

**Table 2:** Clinical studies on the administration of probiotics or pharmaceuticals to eradicate protist infections.

Reference	Year	Study	Symptoms	Treatment	Results
[18]	2006	*S. boulardii* and infection due to *Giardia lamblia*	Enrolled participants showed presence of *G. lamblia* trophozoites or cysts in stool specimens	10 days Group 1: Metronidazole 750 mg tid. 250 mg *S. boulardii* bid. 10 days Group 2: Metronidazole 750mg tid. Placebo bid.	End of week 2, *G. lamblia* cysts detected in 17.1% of Group 2, but none in Group 1. 100% of Group 1 had clearance of microscopic findings 82.8% of Group 2 had clearance of microscopic findings
[19]	2011	Efficacy of *S. boulardii* or metronidazole in symptomatic children with *Blastocystis hominis* infection	Gastrointestinal symptoms: abdominal pain, diarrhea, nausea-vomiting, flatulence Positive stool examination for *Blastocystis hominis*	Group A*: Saccharomyces boulardii* 250 mg bid. Group B: metronidazole 30 mg/kg bid. Group C: no treatment	On day 15 clinical cure observed at: 77.7% in Group A 66.66% in Group B 40% in Group C On day 15 disappearance of cysts from stool was: 80% in Group B 72.2% in Group A 26.6% in Group C
[20]	2009	Efficacy of *S. boulardii* in children with acute bloody diarrhea caused by amebiasis	Acute bloody diarrhea Presence of *Entameba histolytica* cysts in stool	Metronidazole given to both groups Intervention group: 250 mg of *S. boulardii* bid.	Duration of bloody diarrhea was significantly longer in control group Day 5, all amebic cysts disappeared in children in intervention group
[21]	2004	Resolution of *cryptosporidiosis* with probiotic treatment—Case Study of 12 y.o. coeliac sufferer.	Four-month episode of abdominal pain, flatulence, nausea and lethargy. Stool sample revealed *cryptosporidium* oocysts Asymptomatic on a gluten-free diet.	Patient received *Lactobacillus GG* 10^9^ units/day and *Lactobacillus casei Shirota* 6.5 × 10^9^ units/day	Within 10 days, nausea and diarrhea completely resolved and abdominal pain was substantially reduced. Repeat stool sample four weeks following the start of probiotic treatment was clear of *cryptosporidium* oocysts.
[22]	2003	Efficacy *Saccharomyces boulardii* with antibiotics in acute amoebiasis	Patients with acute intestinal amoebiasis with clinical manifestations of acute mucus bloody diarrhea, and amebic trophoxoites engulfing RBCs found in stool specimens	Group 1: Metronidazole 750 mg tid + Iodoquinol 630 mg tid for 10 days Group 2: *Saccharomyces boulardii* 250 mg tid + Metronidazole 750 mg tid + Iodoquinol 630 mg tid for 10 days	At Week 4, amebic cysts were detected in five cases of Group 1, but none in Group 2. Duration of symptoms was significantly less in Group 2 compared to Group 1.
